# A novel analytic approach for outcome prediction in diffuse large B-cell lymphoma by [^18^F]FDG PET/CT

**DOI:** 10.1007/s00259-021-05572-0

**Published:** 2021-10-15

**Authors:** Xiaohui Zhang, Lin Chen, Han Jiang, Xuexin He, Liu Feng, Miaoqi Ni, Mindi Ma, Jing Wang, Teng Zhang, Shuang Wu, Rui Zhou, Chentao Jin, Kai Zhang, Wenbin Qian, Zexin Chen, Cheng Zhuo, Hong Zhang, Mei Tian

**Affiliations:** 1 Department of Nuclear Medicine and PET Center, The Second Affiliated Hospital of Zhejiang University School of Medicine, 88 Jiefang Road, Hangzhou, 310009 Zhejiang China; 2 Key Laboratory of Medical Molecular Imaging of Zhejiang Province, Hangzhou, China; 3 Institute of Nuclear Medicine and Molecular Imaging of Zhejiang University, Hangzhou, China; 4 PET-CT Center, Fujian Medical University Union Hospital, Fuzhou, China; 5 Department of Medical Oncology, The Second Affiliated Hospital of Zhejiang University School of Medicine, Hangzhou, China; 6 Department of Hematology, The Second Affiliated Hospital of Zhejiang University School of Medicine, Hangzhou, China; 7 Department of Clinical Epidemiology & Biostatistics, The Second Affiliated Hospital of Zhejiang University School of Medicine, Hangzhou, China; 8 College of Information Science & Electronic Engineering, Zhejiang University, Hangzhou, China; 9 Key Laboratory for Biomedical Engineering of Ministry of Education, Zhejiang University, Hangzhou, China; 10 College of Biomedical Engineering & Instrument Science, Zhejiang University, Hangzhou, China

**Keywords:** Positron emission tomography (PET), Glucose metabolism, Diffuse large B-cell lymphoma, Prognosis, Radiomics

## Abstract

**Purpose:**

This study aimed to develop a novel analytic approach based on 2-deoxy-2-[^18^F]fluoro-D-glucose positron emission tomography/computed tomography ([^18^F]FDG PET/CT) radiomic signature (RS) and International Prognostic Index (IPI) to predict the progression-free survival (PFS) and overall survival (OS) of patients with diffuse large B-cell lymphoma (DLBCL).

**Methods:**

We retrospectively enrolled 152 DLBCL patients and divided them into a training cohort (*n* = 100) and a validation cohort (*n* = 52). A total of 1245 radiomic features were extracted from the total metabolic tumor volume (TMTV) and the metabolic bulk volume (MBV) of pre-treatment PET/CT images. The least absolute shrinkage and selection operator (LASSO) algorithm was applied to develop the RS. Cox regression analysis was used to construct hybrid nomograms based on different RS and clinical variables. The performances of hybrid nomograms were evaluated using the time-dependent receiver operator characteristic (ROC) curve and the Hosmer–Lemeshow test. The clinical utilities of prediction nomograms were determined via decision curve analysis. The predictive efficiency of different RS, clinical variables, and hybrid nomograms was compared.

**Results:**

The RS and IPI were identified as independent predictors of PFS and OS, and were selected to construct hybrid nomograms. Both TMTV- and MBV-based hybrid nomograms had significantly higher values of area under the curve (AUC) than IPI in training and validation cohorts (all *P* < 0.05), while no significant difference was found between TMTV- and MBV-based hybrid nomograms (*P* > 0.05). The Hosmer–Lemeshow test showed that both TMTV- and MBV-based hybrid nomograms calibrated well in the training and validation cohorts (all *P* > 0.05). Decision curve analysis indicated that hybrid nomograms had higher net benefits than IPI.

**Conclusion:**

The hybrid nomograms combining RS with IPI could significantly improve survival prediction in DLBCL. Radiomic analysis on MBV may serve as a potential approach for prognosis assessment in DLBCL.

**Trial registration:**

NCT04317313. Registered March 16, 2020. Public site: https://clinicaltrials.gov/ct2/show/NCT04317313

**Supplementary Information:**

The online version contains supplementary material available at 10.1007/s00259-021-05572-0.

## Introduction

Diffuse large B-cell lymphoma (DLBCL) represents the most common type of lymphoid neoplasm [1]. Since over 30% of patients experience disease progression or relapse, early identification of high-risk patients is important for patient management [2]. Over the past two decades, the International Prognostic Index (IPI) has been recognized as a prognostic model, which is based on the properties of several clinical factors including age, Ann Arbor stage, extranodal involvement, serum lactate dehydrogenase (LDH) level, and performance status [3]. However, IPI is not suitable for predicting refractory disease, which might be due to its lack of information on intratumoral functional and metabolic profiles [4, 5].

Positron emission tomography/computed tomography (PET/CT) with 2-deoxy-2-[^18^F]fluoro-D-glucose ([^18^F]FDG) is a representative of molecular imaging and transpathology [6], which has been applied as a routine imaging tool for staging and response assessment of lymphoma [7]. Several studies have indicated that PET semi-quantitative parameters, particularly maximum standardized uptake value (SUVmax), total metabolic tumor volume (TMTV), and total lesion glycolysis (TLG), might be independent prognostic factors in DLBCL [8–10]. However, those parameters are only used to evaluate the gross tumor metabolism, which cannot fully depict the subtle metabolic heterogeneity within a targeted lesion. Recently, PET-based radiomics has been introduced as an innovative image analysis that can capture intratumoral metabolic heterogeneity and allow accurate prediction of clinical outcome in various malignancies, such as breast cancer, non-small cell lung cancer, and lymphoma [11–13]. Studies have shown that several single radiomic features, including long-zone high gray-level emphasis, and skeletal textural feature SkewnessH, were significant predictors of survival in DLBCL [14, 15]. Other literatures reported that the combination of multiple radiomic features, which is often defined as the radiomic signature (RS), may hold higher prognostic value than the single feature [16, 17]. In addition, RS combined with clinical or genomic data can produce robust and improved medical decision-making [18, 19]. However, to the best of our knowledge, the RS based on [^18^F]FDG PET/CT for prognosis assessment of DLBCL has not yet been described. Furthermore, it remains unclear whether PET-based RS could add more prognostic values to the IPI in DLBCL.

We hypothesized that RS combined with IPI score could help improve the prognosis assessment of DLBCL patients. Therefore, for the first time, this study investigated the prognostic value of the RS combined with IPI score in predicting the survival of DLBCL patients by [^18^F]FDG PET/CT.

## Materials and methods

### Study population

We retrospectively enrolled patients with newly diagnosed DLBCL between July 2013 and July 2019 in The Second Affiliated Hospital of Zhejiang University School of Medicine. The inclusion criteria were (1) histopathologically confirmed DLBCL, (2) over 18 years old, (3) underwent pre-treatment [^18^F]FDG PET/ CT, and (4) initial treatment with R-CHOP (rituximab, cyclophosphamide, doxorubicin, vincristine, and prednisone) or R-EPOCH (rituximab, etoposide, prednisone, vincristine, cyclophosphamide, and doxorubicin). Patients were excluded if they had coexistent central nervous system lymphoma or other malignancies, or had an incomplete follow-up. In total, 152 patients were enrolled, and divided into a training cohort (*n* = 100) and a validation cohort (*n* = 52) according to the time of enrollment. The flowchart of patient enrollment is shown in Supplemental Fig. 1.

Clinical variables including gender, age at diagnosis, cell of origin, performance status, B symptoms, Ann Arbor stage, serum LDH level, serum β2-microglobulin (β2-MG) level, extranodal involvement, and treatment regimens of each patient were recorded. The IPI score was calculated as described previously [3]. This study was approved by the institutional review board, and the requirement to obtain written informed consent was waived (Approval Number: 2019–350).

### Patient treatment and follow-up

Patients were initially treated with standard first-line chemotherapy every 21 days. Response to treatment was assessed according to the Lugano classification [20]. Those with no response or progressive disease then received involved-site radiotherapy or autologous stem cell transplantation.

The follow-ups were performed every 3 months after the completion of treatment, and ended in Jul 2021. The endpoints included overall survival (OS) (defined as the period from the initial diagnosis to the death from any cause) and progression-free survival (PFS) (defined as the period from the initial diagnosis to the progression, relapse, or death from any cause).

### PET/CT imaging protocol

All images were acquired and reconstructed according to the European Association of Nuclear Medicine (EANM) guidelines version 2.0 [21]. Patients were fasted for at least 6 h and had a blood glucose level below 200 mg/dL before the PET/CT examination. PET/CT imaging was performed at a median uptake time of 67 min (range, 53–81 min) after intravenous injection of [^18^F]FDG (3.7 MBq/kg). Patients were scanned on a PET/CT scanner (Biograph mCT, Siemens Medical Solutions) with 5 min per bed position. A low-dose CT scan (120 kVp; 40–100 mAs; 5 mm slice thickness) was performed from the upper thigh to the base of the skull, followed by a PET scan. PET images were reconstructed with 4.07 × 4.07 × 3 mm^3^ voxels using CT-based attenuation correction by Siemens-specific TrueX algorithm.

All standardized uptake values (SUVs) were normalized for body weight and corrected to the uptake time of 60 min throughout the study. The mean standardized uptake value (SUVmean) of the liver should be between 1.3 and 3.0, consistent with the EANM guidelines [21]. To ensure the accuracy and reproducibility of SUV measurements, a set of quality control (QC) procedures are undertaken, including daily QC, calibration, and cross-calibration. Both daily QC and calibration are performed using a ^68^Ge cylinder with a known radioactive concentration according to the manufacturer’s protocol. The cross-calibration and normalization with time alignment are performed to evaluate the SUV bias according to the manufacturer’s recommendations.

### PET image segmentation and feature extraction

**Fig. 1:**
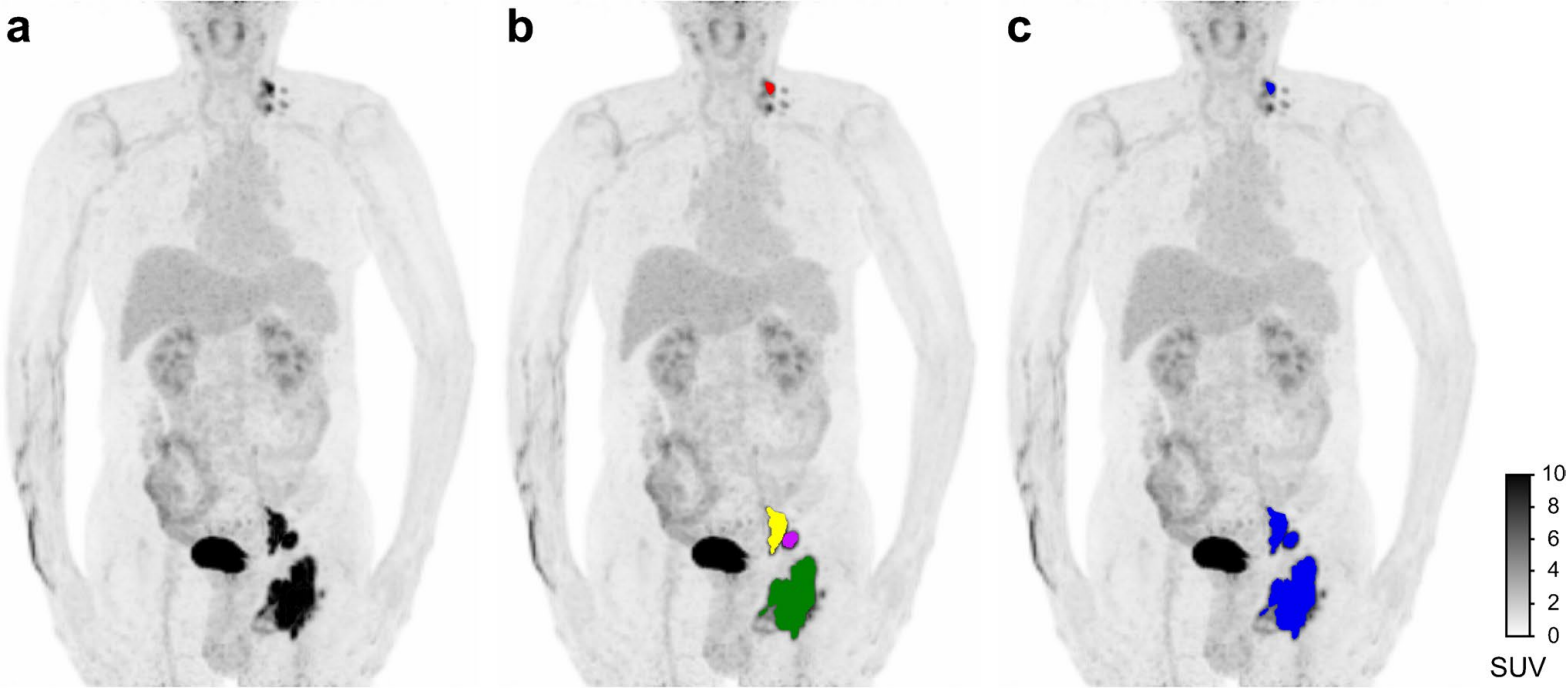
Representative [^18^F]FDG PET images of metabolic bulk volume (MBV) and total metabolic tumor volume (TMTV) delineation. **a** Anterior maximum intensity projection image. **b** The VOIs of cervical (red), iliac (yellow and purple), and inguinal (green) lymph nodes were semiautomatically delineated using the 41% SUVmax threshold method. The VOI of inguinal lymph node (green) represents the MBV. **c** TMTV (blue) was constructed using the “save all in one” function in LIFEx

PET images were analyzed by two experienced nuclear medicine physicians who were blind to the patients’ outcome. The volumes of interest (VOIs) were semiautomatically delineated using the LIFEx software (version 6.30, https://www.lifexsoft.org/index.php) with a fixed threshold of 41% SUVmax [21]. To reduce the influence of partial volume effects, lesions with a minimal diameter of 2 cm on CT or a minimum metabolic volume of 4.2 cm^3^ (if lesion was not apparent on CT) were selected [22]. Bone marrow was considered involved if focal or multifocal lesions presented higher uptake than the liver [23]. For each patient, SUVmax, TMTV, and TLG were recorded. In addition, the metabolic bulk volume (MBV) defined as the metabolic volume of the largest lesion was also recorded [24]. Delineations of MBV and TMTV in LIFEx software are shown in Fig. 1. To assess the time-varying effect on SUV measurements, liver SUVmean and SUVmax were compared between the training and validation cohorts, and also between pre-treatment and end-of-treatment PET scans for patients who underwent end-of-treatment PET/CT evaluation, using a 1.2 cm diameter VOI in posterior right liver lobe, as previously reported [25].

A total of 1245 radiomic features from TMTV and MBV were extracted via PyRadiomics software [26], followed by *z*-score normalization. Feature extraction on TMTV was performed according to a previous literature [13]. Specifically, TMTV was constructed by using the “save all in one” function in LIFEx, and the radiomic features were extracted across the entire metabolic tumor volume. Detailed descriptions of the extracted features are presented in Supplemental Table 1.

### Feature selection and RS construction

Intra-class correlation coefficients (ICCs) were used to evaluate the inter- and intra-observer agreement for 3 conventional and 1245 radiomic features [27]. Features with both inter- and intra-observer ICCs of over 0.75 were retained. The least absolute shrinkage and selection operator (LASSO) Cox regression algorithm with tenfold cross-validation was applied to select the optimal features with non-zero coefficients in the training cohort [28].

The RS based on MBV (MBV-RS) and TMTV (TMTV-RS) were developed through linearly combining the selected features weighted by their corresponding LASSO coefficients. The cut-off values of the RS were identified by X-tile software (version 3.6.1, Yale University).

### Construction of hybrid nomograms

Univariate Cox regression analysis was performed to investigate the prognostic values of the RS and clinical variables. All significant variables were then enrolled into a multivariate Cox regression. MBV- or TMTV-based hybrid nomograms for PFS and OS prediction (MBV-HN_PFS_, TMTV-HN_PFS_, MBV-HN_OS_, and TMTV-HN_OS_) were then established on the basis of the regression coefficient of each variable that remained significant in the multivariate Cox analysis [29]. Based on the established nomogram, a risk score was calculated for each patient in the training cohort. An optimal cut-off point of the risk score was determined by maximized Youden index to stratify patients into low-risk or high-risk groups [30].

### Model performance assessment

Time-dependent receiver operator characteristic (ROC) curve analysis was conducted to investigate the predictive accuracies of RS, clinical variables, and hybrid nomograms [31]. Sensitivity, specificity, positive predictive value, and negative predictive value were calculated accordingly. The cut-off values of RS, clinical variables, and risk score of hybrid nomograms evaluated in the training cohort were applied to the validation cohort. The area under the curves (AUCs) between the hybrid nomograms and clinical variables were compared by using the DeLong test [32]. The Hosmer–Lemeshow test was used to evaluate the goodness-of-fit of the hybrid nomograms [33]. The decision curve analysis (DCA) was applied to determine the clinical utilities of the hybrid nomograms by quantifying the net benefits under different threshold probabilities in the whole cohort [34].

### Statistical analysis

All statistical analyses were performed by using R (version 3.6.1, http://www.r-project.org) and SPSS software (version 25.0, IBM). The differences in clinical characteristics between the training and validation cohorts were assessed by using the chi-square test and independent *t*-test, where appropriate. Survival functions were estimated by Kaplan–Meier analysis, and survival distributions were compared by using log-rank test. A *P* value of < 0.05 was considered statistically significant.

## Results

### Patient characteristics and outcome

The patients’ characteristics are summarized in Table 1. No significant difference in clinical characteristics was observed between the training and the validation cohort (*P* = 0.062–0.888). No time-varying effect on SUV measurement was identified (Supplemental Table 2). The median follow-up period of the whole cohort was 42.5 months (range 4–96 months). By the end of follow-up, 49 patients (32.2%) had a PFS event (with a median of 11.5 months), while 41 patients (27%) died (with a median of 14 months).

**Table 1:** Clinical characteristics of enrolled patients

Patient characteristics	Training cohort (*n* = 100)	Validation cohort (*n* = 52)	P *
Age (mean ± SD) (years)	57.8 ± 14.6	59.4 ± 15.7	0.533
Gender			0.494
Male	48	28	
Female	52	24	
LDH			0.586
Normal	45	21	
Elevated	55	31	
β2-MG			0.367
Normal	67	31	
Elevated	33	21	
Ann Arbor stage			0.261
I–II	38	15	
III–IV	62	37	
Performance status			0.062
< 2	76	32	
≥ 2	24	20	
Extranodal sites			0.099
< 2	71	30	
≥ 2	29	22	
B symptoms			0.188
Yes	28	20	
No	72	32	
IPI score			0.664
≤ 2	54	30	
> 2	46	22	
Cell of origin			0.888
GCB	28	14	
Non-GCB	72	38	
Treatment			0.729
Chemotherapy alone	72	41	
Chemotherapy + ISRT	25	10	
Chemotherapy + ASCT	3	1	
Chemotherapy regimens			
R-CHOP	95	48	0.760
R-EPOCH	5	4	
Endpoint			
2-year PFS rate (%)	75.0	71.2	0.271
2-year OS rate (%)	80.0	76.9	0.352

*LDH* lactate dehydrogenase, *β2-MG* β2-microglobulin, *IPI* International Prognostic Index, *GCB* germinal center B-cell like, *ISRT* involved-site radiotherapy, *ASCT* autologous stem cell transplantation, *PFS* progression-free survival, *OS* overall survival

^*^P value was calculated by independent *t*-test for continuous variables, chi-square test for categorical variables, and log-rank test for survival rates

### Relationship between MBV and TMTV

The location of the tumor bulk is summarized in Supplemental Table 3. MBV was significantly correlated with TMTV (Pearson’s correlation coefficient *r* = 0.778; *P* < 0.0001) (Supplemental Fig. 2). No significant difference was found in AUCs between MBV and TMTV for predicting PFS and OS in the training (*P* = 0.161 and *P* = 0.526, respectively) nor the validation cohort (*P* = 0.967 and *P* = 0.940, respectively) (Supplemental Fig. 3).

The optimal cut-off values of MBV and TMTV were 39.9 cm^3^ and 104.6 cm^3^, respectively. As shown in Supplemental Fig. 4, the overall concordance between MBV and TMTV was 88% (lower MBV and lower TMTV: 42%; higher MBV and higher TMTV: 46%) in training cohort, and 75% (lower MBV and lower TMTV: 28.8%, higher MBV and higher TMTV: 46.2%) in validation cohort. The overall discordance between MBV and TMTV was 12% (lower MBV and higher TMTV: 2%, with 2-year PFS of 50% and 2-year OS of 100%; higher MBV and lower TMTV: 10%, both 2-year PFS and OS were 70%) in training cohort, and 25% (lower MBV and higher TMTV: 3.8%, both 2-year PFS and OS were 100%; higher MBV and lower TMTV: 21.2%, both 2-year PFS and OS were 81.8%) in validation cohort.

### Construction of RS

Of the 1248 features, 1139 MBV-extracted and 1032 TMTV-extracted features had good repeatability (ICCs > 0.75) (Supplemental Table 4). MBV- and TMTV-based feature selection is shown in Supplemental Figs 5 and 6, respectively. None of the conventional PET parameters was retained. Details of the selected features are shown in Supplemental Table 5. The cut-off values of the MBV-RS_PFS_, MBV-RS_OS_, TMTV-RS_PFS_, and TMTV-RS_OS_ were 0.01, − 0.14, − 0.21, and − 0.28, respectively.

### Construction of hybrid nomograms

**Fig. 2:**
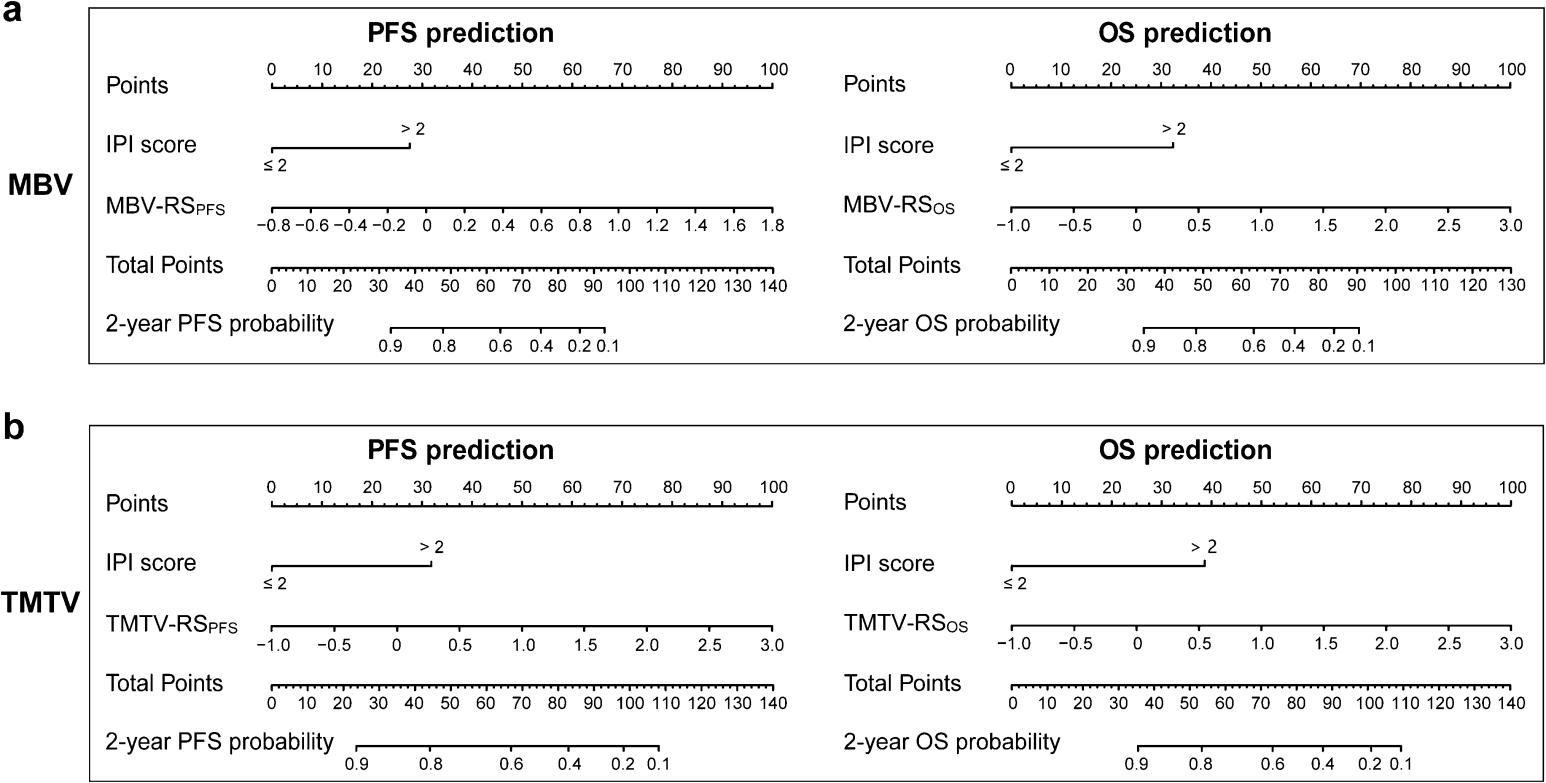
Hybrid nomograms combining radiomic signatures (RS) and IPI score based on **a** MBV and **b** TMTV for PFS and OS prediction

Univariate Cox analysis showed that β2-MG, B symptoms, IPI score, MBV-RS_PFS_, and TMTV-RS_PFS_ were significantly associated with PFS, while IPI score, MBV-RS_OS_, and TMTV-RS_OS_ were significantly associated with OS (Supplemental Table 6). In the multivariate analysis, the RS (MBV-RS_PFS_, TMTV-RS_PFS_, MBV-RS_OS_, and TMTV-RS_OS_) and IPI score were independent predictors of PFS and OS (Supplemental Table 7), and were selected to build the hybrid nomograms based on MBV (Fig. 2a) and TMTV (Fig. 2b). The cut-off values of risk score for MBV-HN_PFS_, MBV-HN_OS_, TMTV-HN_PFS_, and TMTV-HN_OS_ correspond to 51, 69, 54, and 63 total points, respectively.

### Model performance assessment

**Fig. 3:**
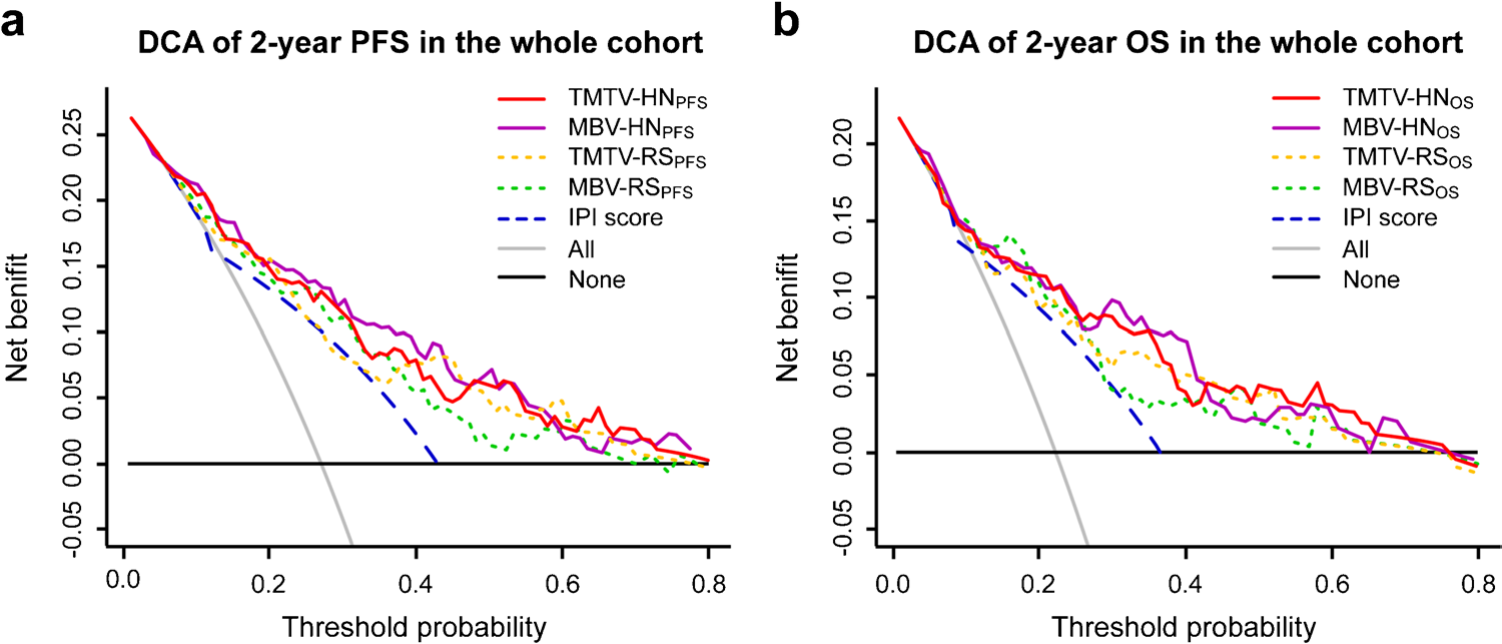
Decision curve analysis (DCA) of **a** PFS and **b** OS for hybrid nomograms (HN), radiomic signatures (RS), and IPI score in the whole cohort

The diagnostic performances of hybrid nomograms, RS, and IPI score are presented in Table 2. For PFS prediction, TMTV-based hybrid nomogram had significantly higher AUCs than the IPI score in both training cohort (0.828 vs. 0.701, *P* < 0.001) and validation cohort (0.783 vs. 0.663, *P* = 0.041). Significant differences of AUC were also found between MBV-based hybrid nomogram and IPI score in both training cohort (0.835 vs. 0.701, *P* < 0.001) and validation cohort (0.787 vs. 0.663, *P* = 0.017). No significant difference was observed between the two hybrid nomograms in the training (*P* = 0.456) nor the validation cohort (*P* = 0.971). There was no significant difference between MBV-RS_PFS_/TMTV-RS_PFS_ and IPI score in the two cohorts (all *P* > 0.05). The Hosmer–Lemeshow test showed that both TMTV- and MBV-based hybrid nomograms calibrated well in the two cohorts (all *P* > 0.05). DCA of 2-year PFS indicated that TMTV- and MBV-based hybrid nomograms had higher net benefits than IPI score (threshold probability over 7% and 6%, respectively) (Fig. 3a).

**Table 2:** Diagnostic performances of hybrid nomograms, radiomic signatures and IPI score

End points	Models	Training cohort							Validation cohort						
		AUC (95% CI)	Sens (%)	Spec (%)	PPV (%)	NPV (%)	DeLong test *P* *	HL test *P*	AUC (95% CI)	Sens (%)	Spec (%)	PPV (%)	NPV (%)	DeLong test *P* *	HL test *P*
**PFS**	Hybrid nomogram														
	TMTV-HN_PFS_	0.828 (0.721–0.916)	80.0	73.3	50.0	91.7	< 0.001	0.389	0.781 (0.647–0.905)	75.0	75.0	57.1	87.1	0.041	0.110
	MBV-HN_PFS_	0.835 (0.729–0.922)	80.0	74.7	51.3	91.8	< 0.001	0.276	0.787 (0.651–0.907)	75.0	77.8	60.0	87.5	0.017	0.431
	Radiomic signature														
	TMTV-RS_PFS_	0.819 (0.724–0.909)	84.0	66.7	45.7	92.6	0.104	0.231	0.748 (0.596–0.886)	81.3	66.7	52.0	88.9	0.285	0.398
	MBV-RS_PFS_	0.806 (0.704–0.898)	84.0	62.7	42.9	92.2	0.455	0.620	0.759 (0.595–0.888)	68.8	80.5	61.1	85.3	0.209	0.548
	IPI score	0.701 (0.592–0.796)	76.0	64.0	41.3	88.9	NA	NA	0.663 (0.512–0.798)	68.8	63.9	45.8	82.1	NA	NA
**OS**	Hybrid nomogram														
	TMTV-HN_OS_	0.818 (0.699–0.912)	80.0	66.3	37.2	93.0	0.005	0.492	0.785 (0.632–0.925)	78.6	71.1	50.0	90.0	0.038	0.188
	MBV-HN_OS_	0.831 (0.723–0.916)	80.0	72.5	42.1	93.5	< 0.001	0.549	0.792 (0.621–0.936)	78.6	76.3	55.0	90.6	0.013	0.259
	Radiomic signature														
	TMTV-RS_OS_	0.803 (0.681–0.898)	85.0	62.5	36.2	94.3	0.563	0.403	0.742 (0.554–0.917)	71.4	73.7	50.0	87.5	0.268	0.219
	MBV-RS_OS_	0.815 (0.719–0.904)	90.0	66.3	40.0	96.4	0.096	0.336	0.757 (0.586–0.906)	78.6	68.4	47.8	89.7	0.192	0.567
	IPI score	0.713 (0.606–0.807)	80.0	62.5	34.8	92.6	NA	NA	0.652 (0.483–0.795)	64.3	65.8	40.9	83.3	NA	NA

*AUC* area under the curve, *CI* confidence interval, *Sens* sensitivity, *Spec* specificity, *PPV* positive predictive value, *NPV* negative predictive value, *HL* Hosmer–Lemeshow, *PFS* progression-free survival, *TMTV* total metabolic tumor volume, *HN* hybrid nomogram, *MBV* metabolic bulk volume, *RS* radiomic signature, *IPI* International Prognostic Index, *NA* not applicable, *OS* overall survival

^*^P value was calculated by comparing AUC with that of the IPI score

For OS prediction, the AUCs of TMTV-based hybrid nomogram were significantly higher than IPI score in both training cohort (0.818 vs. 0.713, *P* = 0.005) and validation cohort (0.789 vs. 0.652, *P* = 0.038). Similar results were also observed between MBV-based hybrid nomogram and IPI score in both training (0.831 vs. 0.713, *P* < 0.001) and validation cohort (0.792 vs. 0.652, *P* = 0.013). There was no significant difference between TMTV- and MBV-based hybrid nomograms in the training (*P* = 0.242) nor the validation cohort (*P* = 0.965). Also, no significant difference was observed between MBV-RS_OS_/TMTV-RS_OS_ and IPI score (all *P* > 0.05). Both TMTV- and MBV-based hybrid nomograms calibrated well in the two cohorts (all *P* > 0.05). TMTV- and MBV-based hybrid nomograms had higher net benefits than IPI score if the threshold probability was over 9% and 4%, respectively (Fig. 3b).

### Survival prediction

**Fig. 4:**
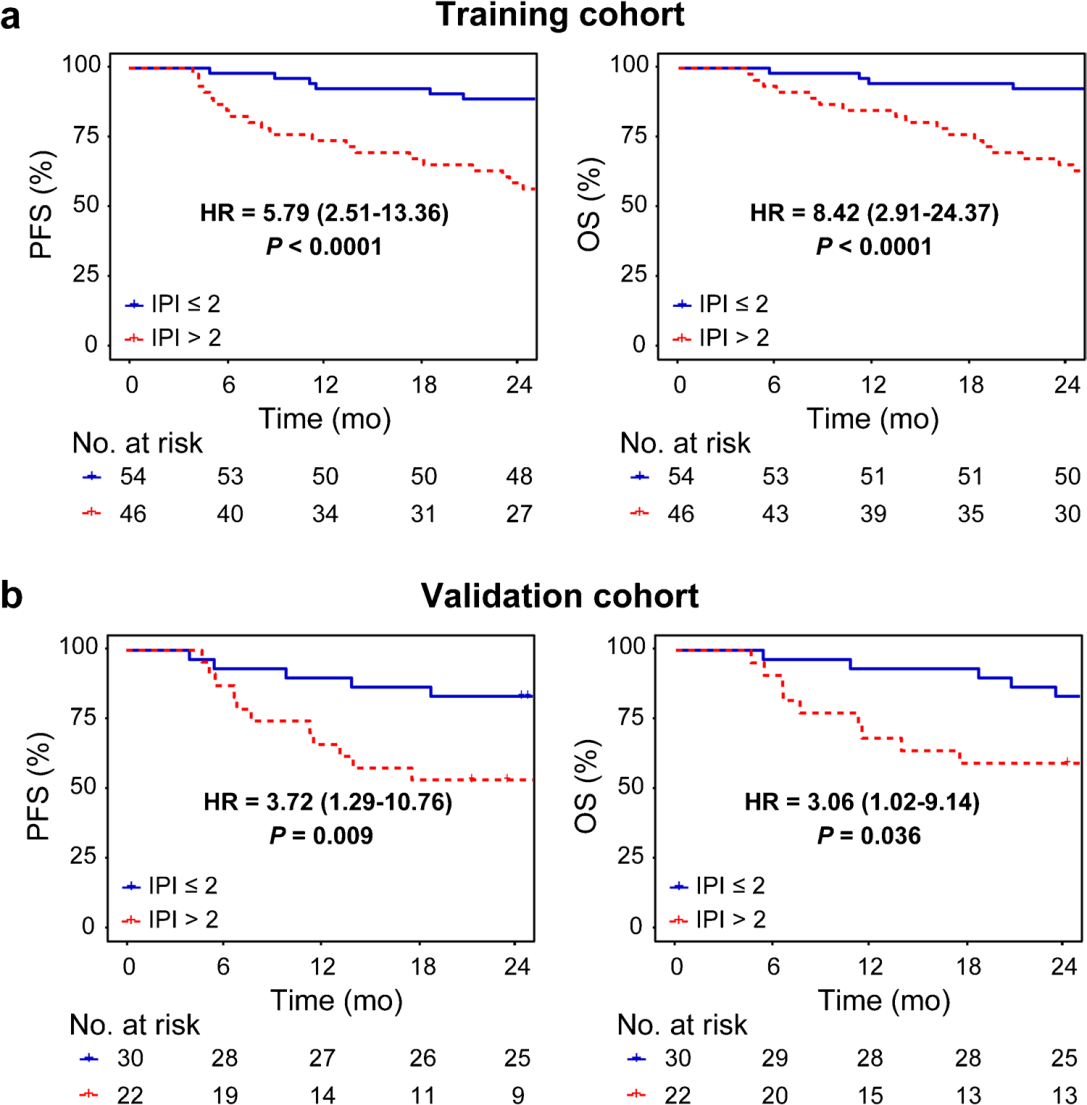
Kaplan–Meier estimates of PFS and OS according to IPI score in **a** the training cohort and **b** the validation cohort. Hazard ratio (HR) with 95% confidence interval and log-rank *P* value are reported

**Fig. 5:**
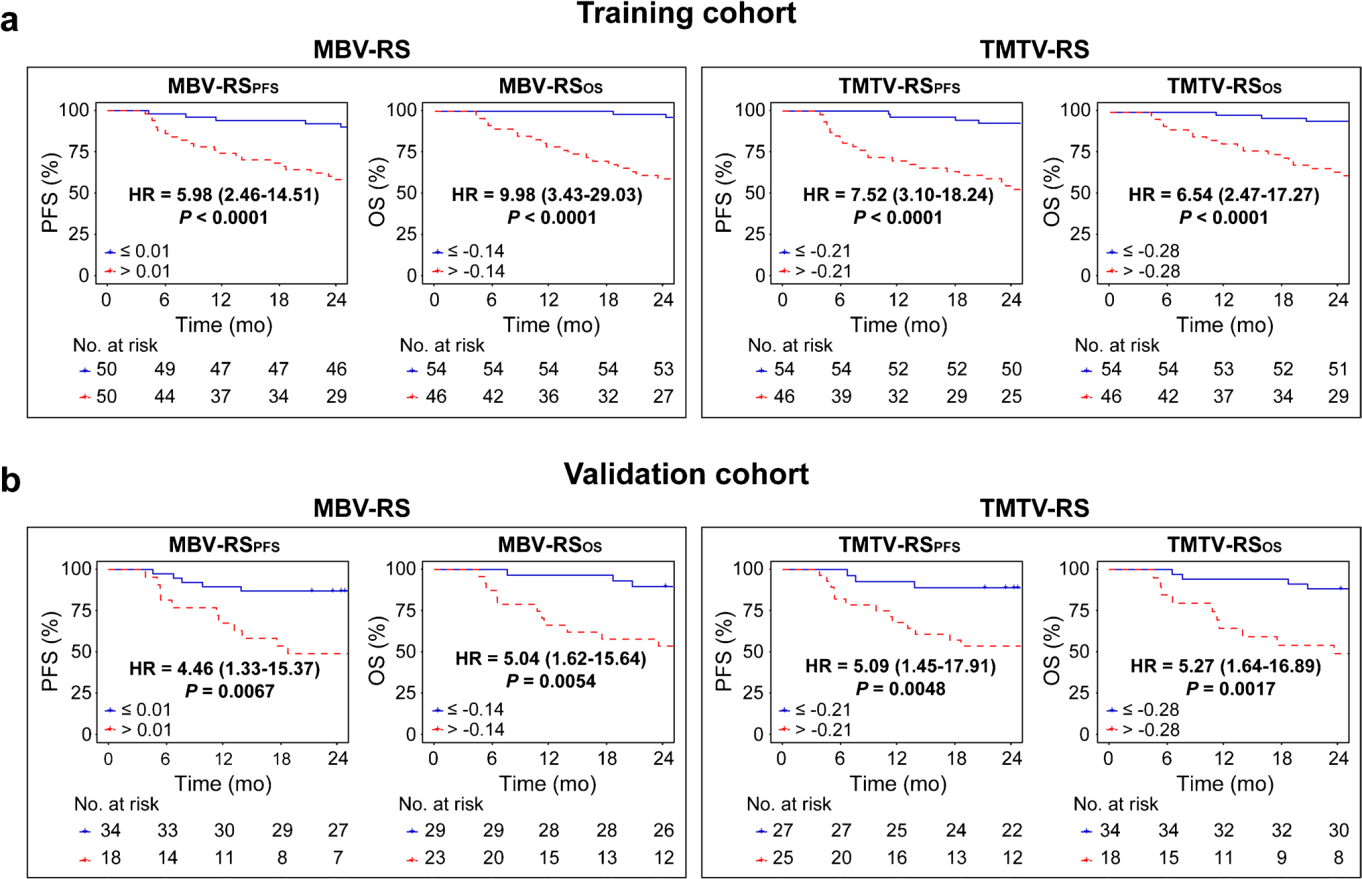
Kaplan–Meier estimates of PFS and OS according to MBV- and TMTV-based radiomic signatures (RS) in **a** the training cohort and **b** the validation cohort. Hazard ratio (HR) with 95% confidence interval and log-rank *P* value are reported

**Fig. 6:**
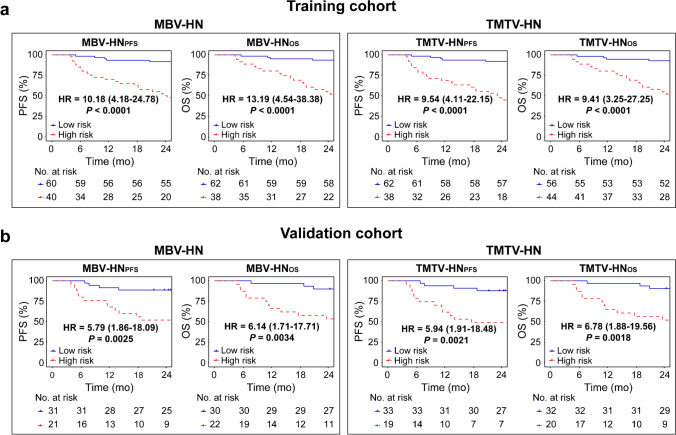
Kaplan–Meier estimates of PFS and OS according to MBV- and TMTV-based hybrid nomograms (HN) in **a** the training cohort and **b** the validation cohort. Hazard ratio (HR) with 95% confidence interval and log-rank *P* value are reported

Kaplan–Meier estimates showed that patients could be stratified into distinct subgroups according to IPI score (Fig. 4) and RS (Fig. 5) (all *P* < 0.05). By combining RS with IPI, we observed that both MBV- and TMTV-based hybrid nomograms demonstrated a more distinct risk stratification than IPI alone, with larger differences between subgroups and improved hazard ratios (all *P* < 0.05) (Fig. 6).

## Discussion

In the present study, we developed RS and hybrid nomograms from pre-treatment [^18^F]FDG PET/CT images for outcome prediction in patients with DLBCL. The RS and IPI were identified as independent predictors of PFS and OS. The hybrid nomograms combining RS with IPI performed better than IPI alone, indicating that the RS could provide incremental prognostic values to the IPI. To the best of our knowledge, this is the first study that combines RS with IPI to assess the intratumoral heterogeneity on pre-treatment [^18^F]FDG PET/CT in DLBCL.

The most important finding of our study was that the RS composed of multiple radiomic features could improve the prognostic value beyond the conventional IPI score. The hybrid nomograms combining RS with IPI could help stratify those high-risk individuals with poorer survival outcomes, achieving significantly higher AUCs and contributing to more distinct risk stratifications than IPI alone. This is consistent with a very recent study indicating that a single radiomic feature run length non-uniformity could provide additional prognostic value to the IPI in DLBCL [35]. However, compared with the run length non-uniformity reported in their study, the RS in our study showed more significant *P* values in the multivariate analysis (e.g., the *P* value of the MBV-based RS for PFS prediction was 0.001). Similarly, another study also identified a single radiomic feature long-zone high gray-level emphasis as an independent predictor of 2-year event-free survival (with a sensitivity of 0.60) [14]. By comparison, the RS in our study showed higher sensitivities for survival prediction (e.g., the MBV-based RS had a sensitivity of 0.84 for PFS prediction in the training cohort). In our study, neither MBV- nor TMTV-based RS showed significantly higher AUCs than IPI score. This observation is in line with previous studies demonstrating that the predictive ability was comparable between RS and clinical variables [36, 37]. A possible explanation is that IPI score and RS possess different properties in phenotyping disease characteristics. IPI score is based only on clinical factors, while RS represents imaging features that reflect the intratumoral metabolic heterogeneity. Since the complex nature and biologic processes of malignancy involve multiple components, taking both clinical and imaging features into account may provide a more comprehensive disease characterization and a better prognostication. In this study, we reported the first attempt to develop hybrid nomograms by combining the PET-based RS with IPI, which was supposed to provide an individualized estimate of survival and could serve as an easy-to-use tool for clinical decision-making. Taken previous findings and our results together, we speculated that PET-based radiomics and IPI could be complementary and synergistic for estimating survival in DLBCL.

Radiomic analysis for lymphoma is challenging, at least in part due to the inter- and intratumoral heterogeneity, and the complexity of isolated lesion segmentation especially when disease is disseminated [38]. In light of these concerns, we performed radiomic analysis on the metabolic volume of the largest lesion, which was defined as MBV in our study, and compared its performance with that based on TMTV. Our results demonstrated that radiomic analysis on MBV and TMTV both perform well in predicting survival, which is in line with previous reports [13, 14]. Moreover, no significant difference was found between the performance of MBV- and TMTV-based hybrid nomograms. As measuring MBV is technically easier and faster than measuring TMTV, our results indicated that radiomic analysis on MBV could be a feasible approach for prognosis assessment in DLBCL.

We also compared the prognostic value of MBV and TMTV. While TMTV has been commonly reported as a potential prognostic indicator in DLBCL [9, 39], very few studies have focused on the prognostic effect of MBV. A recent study suggested that MBV was an independent predictor of OS and had a strong correlation with TMTV [24]. Consistently, ROC analysis in our study showed no significant difference between MBV and TMTV in survival prediction, suggesting that MBV holds prognostic value as TMTV does. Besides, for 25% of patients in validation cohort who had discordance in MBV and TMTV, MBV accurately predicted the outcome regardless of TMTV, which was in accordance with the previous finding that MBV might have greater influence on survival than TMTV [24], and indicated that MBV could be a surrogate marker of TMTV.

Our results showed that conventional PET parameters including SUVmax, TMTV, and TLG were not retained for model construction. However, these parameters were reported to be predictive of survival in DLBCL [8–10]. This discrepancy may be attributed to the differences in the methods employed for feature selection and model building. In our study, we applied the LASSO Cox regression algorithm which is considered suitable for screening high-dimensional features that are most strongly associated with patient outcome and avoiding overfitting [40]. As shown in our results, only radiomic features were finally selected via this algorithm, indicating that radiomic features were correlated with tumor volume and might provide more accurate prognostic information than conventional PET parameters in DLBCL.

In our study, we applied the 41% SUVmax method which has been recommended by the EANM and identified as an effective approach for prognosis assessment in DLBCL [21, 41, 42]. The results of the ICC analysis showed that the majority of assessed features had good intra- and inter-observer agreement (ICC > 0.75), which is consistent with a recent study demonstrating that the 40% SUVmax method could improve the repeatability of most radiomic features [43]. However, it has been revealed that the radiomic features could be influenced by different segmentation methods [44]. Future studies are required to use multiple segmentation and explore optimized methods through more advanced, deep learning techniques [45, 46].

There are some limitations to this study. First, protein expressions and gene arrangements of MYC and BCL-2 are acknowledged prognostic factors but are not evaluated in our study due to the unavailability of these data from all patients. Second, one should be cautious when extrapolating these findings as this is a retrospective single-center study with a relatively small sample size. Therefore, our results need to be further validated in prospective multi-center studies involving a larger cohort of patients.

## Conclusion

In this study, we developed a novel analytic approach based on RS and IPI score for predicting the outcome of patients with DLBCL by [^18^F]FDG PET/CT, which showed significant predictive performance. MBV-based radiomic analysis may serve as a potential approach for prognosis assessment in DLBCL.

## Supplementary Information

Below is the link to the electronic supplementary material.

Supplementary file1 (DOCX 1427 kb)

## Data Availability

The data sets generated and analyzed during the current study are available from the corresponding author on reasonable request.
