# Supplementary material for: A novel analytic approach for outcome prediction in diffuse large B-cell lymphoma by [18F]FDG PET/CT
Source: Eur J Nucl Med Mol Imaging. 2021 Oct 15;49(4):1298–310. doi: 10.1007/s00259-021-05572-0 (PMC8921097; doi:10.1007/s00259-021-05572-0)
Supplement: Supplementary file 1 — Supplementary file1 (DOCX 1427 kb) [file 259_2021_5572_MOESM1_ESM.docx]

**A novel analytic approach for outcome prediction in diffuse large B-cell lymphoma by [^18^F]FDG PET/CT**

Xiaohui Zhang ^1-3#^, Lin Chen^1-3#^, Han Jiang^4#^, Xuexin He^5^, Liu Feng^1-3^, Miaoqi Ni^1-3^, Mindi Ma^1-3^, Jing Wang^1-3^, Teng Zhang^1-3^, Shuang Wu^1-3^, Rui Zhou^1-3^, Chentao Jin^1-3^, Kai Zhang^1^, Wenbin Qian^6^, Zexin Chen^7^, Cheng Zhuo^8^, Hong Zhang^1-3,9,10^* and Mei Tian^1-3, 9^*

1. Department of Nuclear Medicine and PET Center, The Second Affiliated Hospital of Zhejiang University School of Medicine, Hangzhou, China
2. Key Laboratory of Medical Molecular Imaging of Zhejiang Province, Hangzhou, China
3. Institute of Nuclear Medicine and Molecular Imaging of Zhejiang University, Hangzhou, China
4. PET-CT Center, Fujian Medical University Union Hospital, Fuzhou, China
5. Department of Medical Oncology, The Second Affiliated Hospital of Zhejiang University School of Medicine, Hangzhou, China
6. Department of Hematology, The Second Affiliated Hospital of Zhejiang University School of Medicine, Hangzhou, China
7. Department of Clinical Epidemiology & Biostatistics, The Second Affiliated Hospital of Zhejiang University School of Medicine, Hangzhou, China
8. College of Information Science & Electronic Engineering, Zhejiang University, Hangzhou, China
9. Key Laboratory for Biomedical Engineering of Ministry of Education, Zhejiang University, Hangzhou, China
10. College of Biomedical Engineering & Instrument Science, Zhejiang University, Hangzhou, China

*Corresponding authors:

Prof. Mei Tian, E-mail: [meitian@zju.edu.cn](mailto:meitian@zju.edu.cn)

Prof. Hong Zhang, E-mail: [hzhang21@zju.edu.cn](mailto:hzhang21@zju.edu.cn)


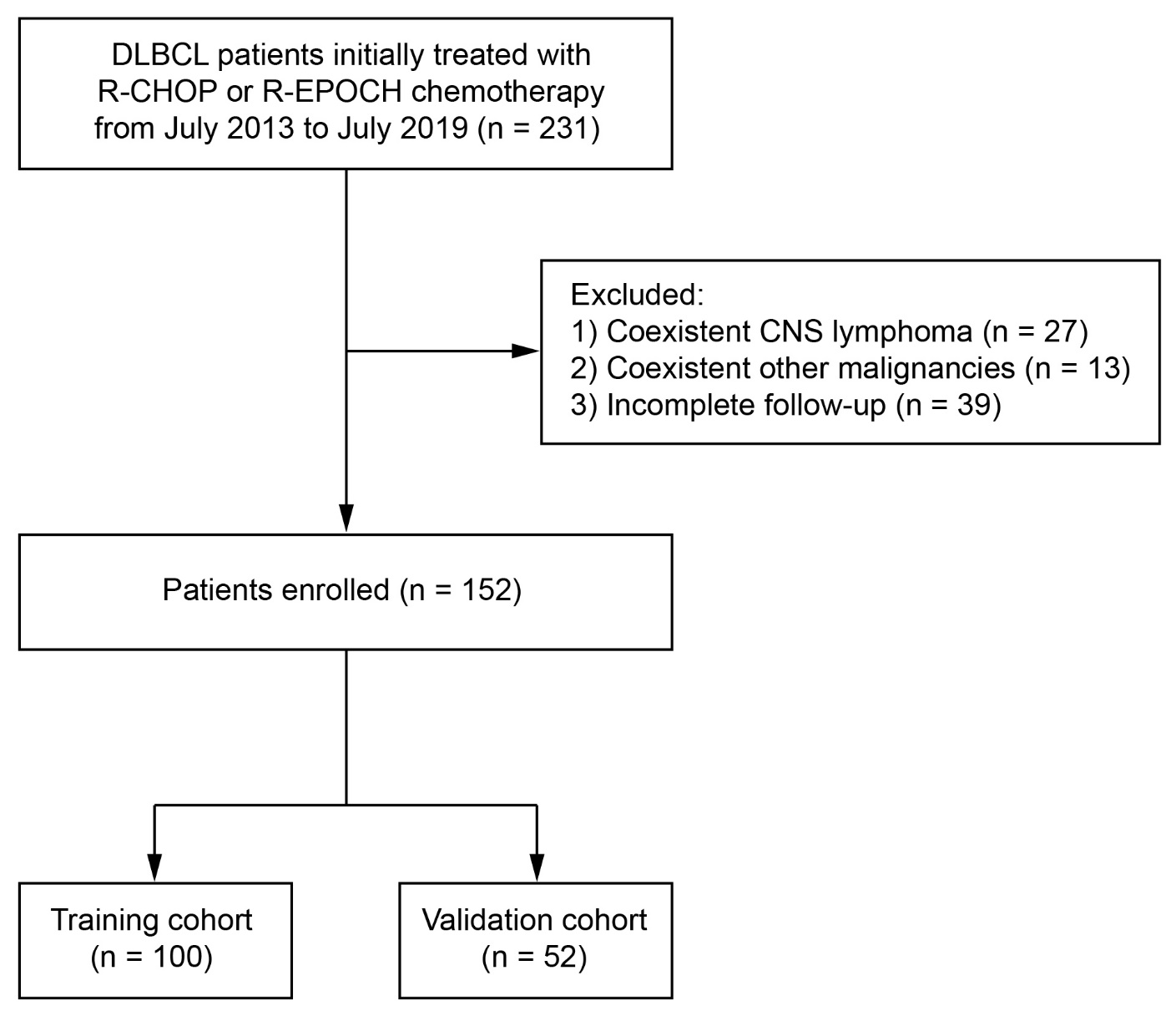


**Supplemental Fig. 1** Flowchart of DLBCL patient enrollment throughout the study. According to the time of enrollment, patients diagnosed between July 2013 and March 2017 formed the training cohort, and those diagnosed between April 2017 and July 2019 formed the validation cohort.


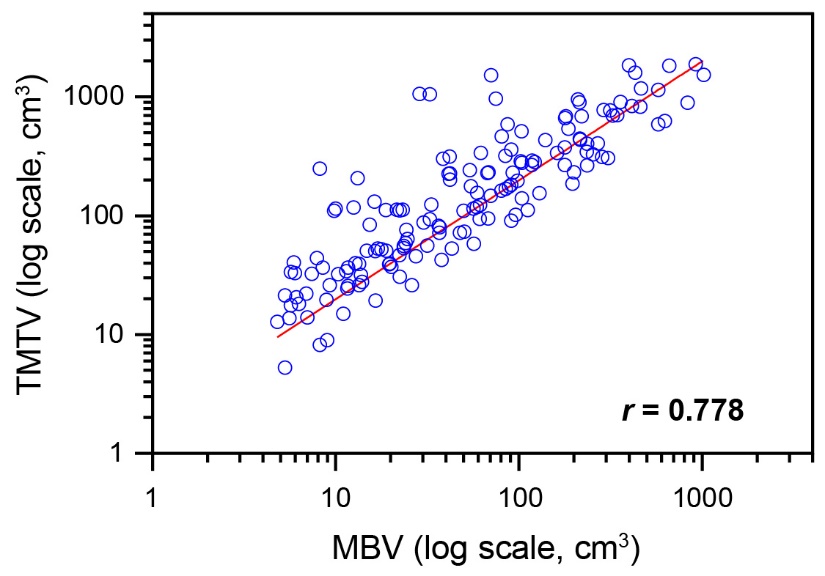


**Supplemental Fig. 2** Relationship between metabolic bulk volume (MBV) and total metabolic tumor volume (TMTV) in the whole cohort.


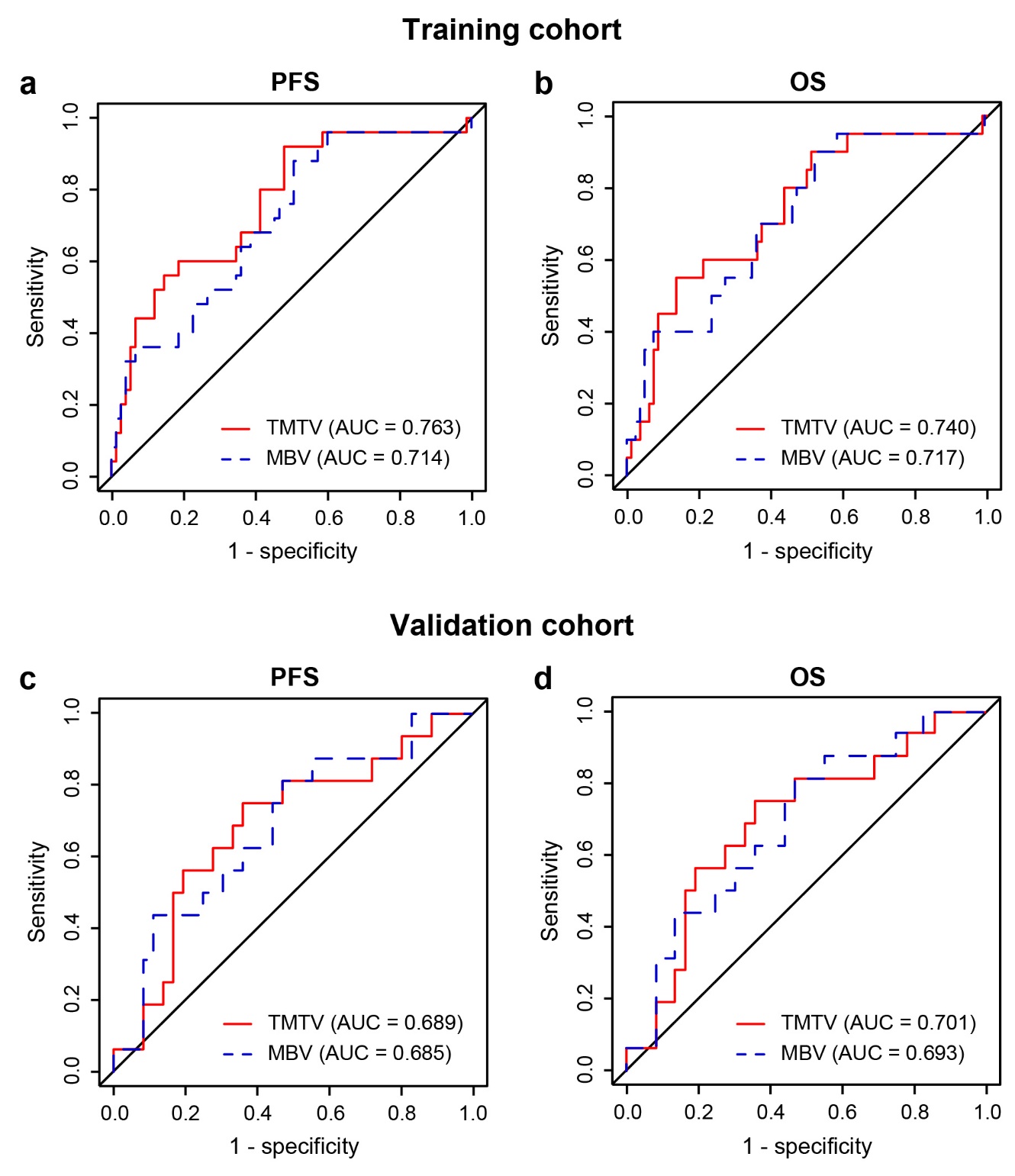


**Supplemental Fig. 3** Time-dependent ROC curves of the metabolic bulk volume (MBV) and total metabolic tumor volume (TMTV) in predicting 2-year PFS and OS. (**a**) ROC curves for PFS in training cohort. (**b**) ROC curves for OS in training cohort. (**c**) ROC curves for PFS in validation cohort. (**d**) ROC curves for OS in validation cohort.


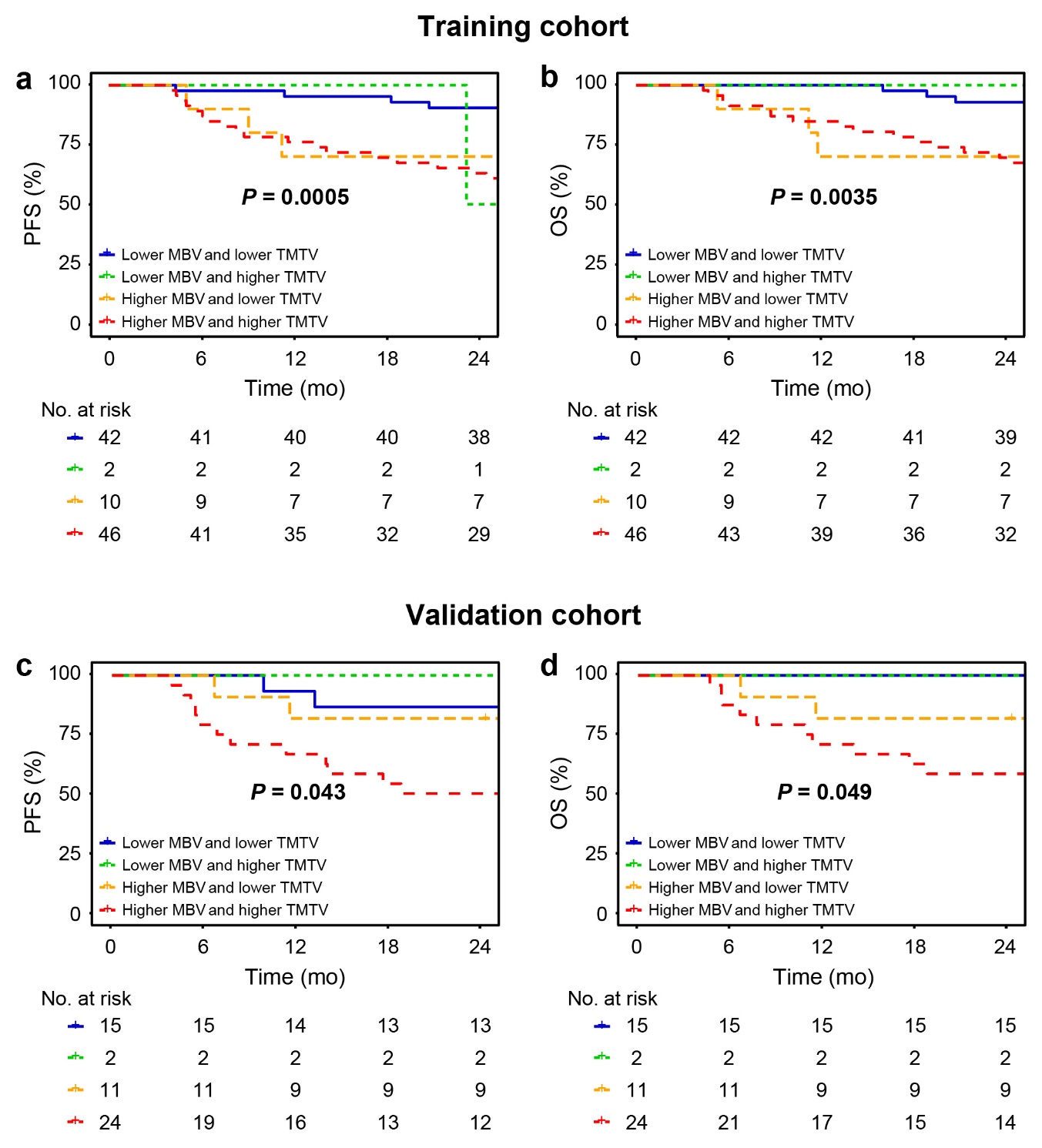


**Supplemental Fig. 4** Kaplan-Meier estimates of PFS and OS according to metabolic bulk volume (MBV) and total metabolic tumor volume (TMTV). (**a**) Survival curves of PFS in training cohort. (**b**) Survival curves of OS in training cohort. (**c**) Survival curves of PFS in validation cohort. (**d**) Survival curves of OS in validation cohort. *P* values according to log-rank test are reported.


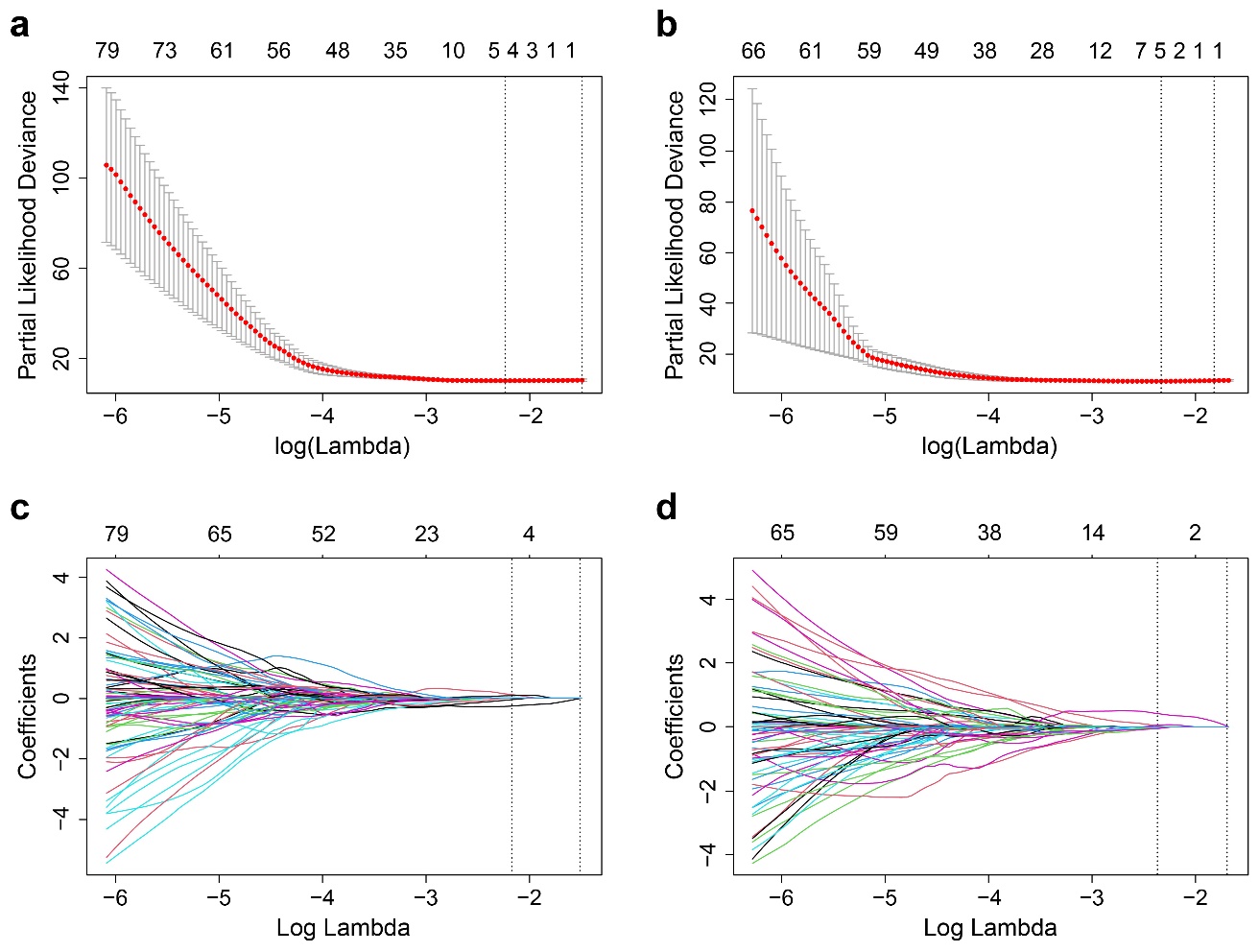


**Supplemental Fig. 5** Selection of radiomic features extracted from the metabolic bulk volume by using the least absolute shrinkage and selection operator (LASSO) Cox regression algorithm. The selected tuning parameter (Lambda) in LASSO model through 10-fold cross-validation were with minimum criteria in predicting (**a**) PFS and (**b**) OS. LASSO coefficient profiles of the radiomic features in predicting (**c**) PFS and (**d**) OS. The dotted vertical lines indicate the optimal values using the minimum criteria.


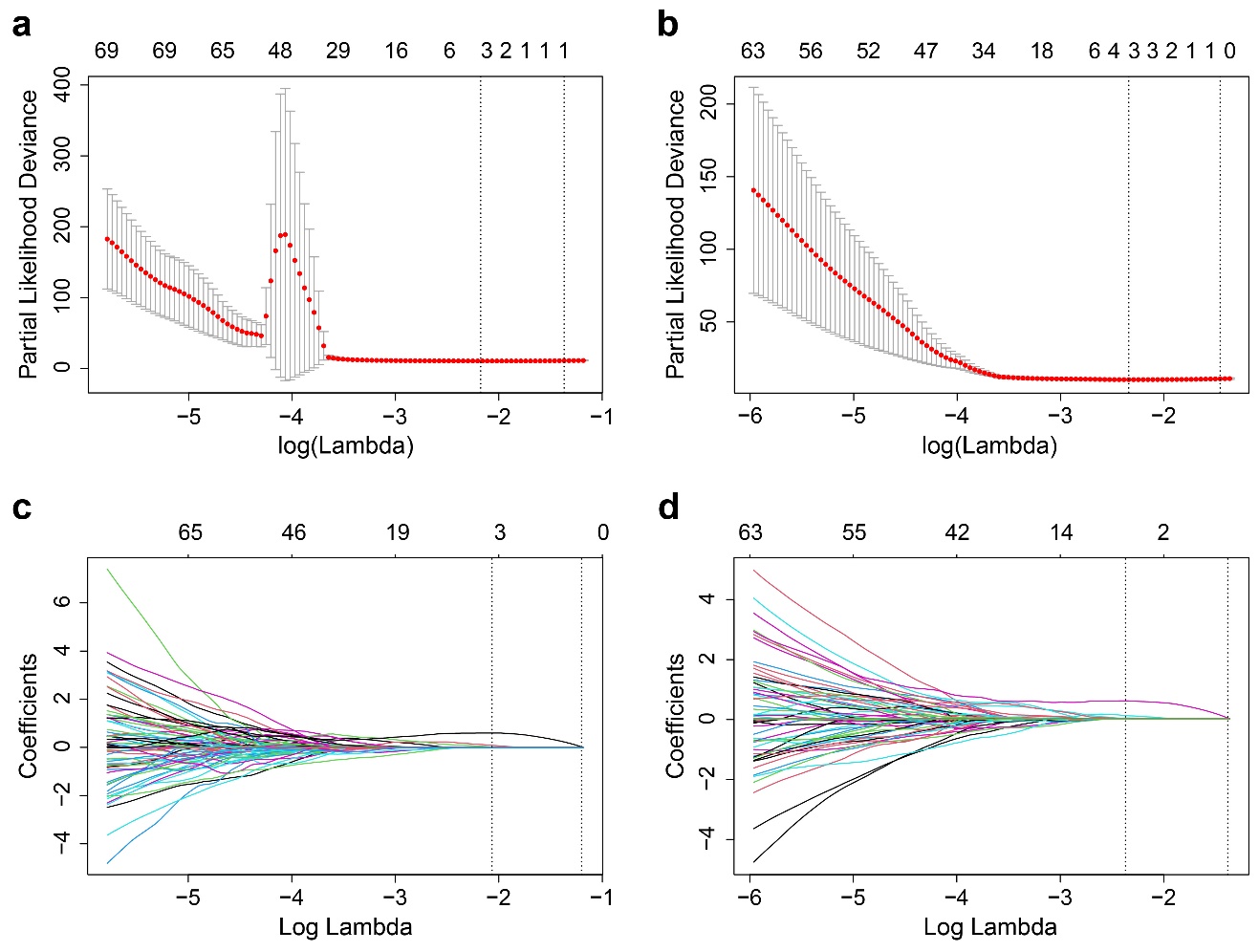


**Supplemental Fig. 6** Selection of radiomic features extracted from the total metabolic tumor volume by using the least absolute shrinkage and selection operator (LASSO) Cox regression algorithm. The selected tuning parameter (Lambda) in LASSO model through 10-fold cross-validation were with minimum criteria in predicting (**a**) PFS and (**b**) OS. LASSO coefficient profiles of the radiomic features in predicting (**c**) PFS and (**d**) OS. The dotted vertical lines indicate the optimal values using the minimum criteria.

**Supplemental Table 1** List of extracted radiomic features

| **Classification** | **Method** | **Feature name** | **Count** |
| --- | --- | --- | --- |
| Shape | - | Elongation | 13 |
| features |  | Flatness |  |
|  |  | Least Axis |  |
|  |  | Major Axis |  |
|  |  | Maximum 2D Diameter Column |  |
|  |  | Maximum 2D Diameter Row |  |
|  |  | Maximum 2D Diameter Slice |  |
|  |  | Maximum 3D diameter |  |
|  |  | Minor Axis |  |
|  |  | Sphericity |  |
|  |  | Surface Area |  |
|  |  | Surface Volume Ratio |  |
|  |  | Volume |  |
|  |  |  |  |
| First-order | Histogram | 10 Percentile | 18 |
| features |  | 90 Percentile |  |
|  |  | Energy |  |
|  |  | Entropy |  |
|  |  | Interquartile Range |  |
|  |  | Kurtosis |  |
|  |  | Maximum |  |
|  |  | Mean |  |
|  |  | Mean Absolute Deviation |  |
|  |  | Median |  |
|  |  | Minimum |  |
|  |  | Range |  |
|  |  | Robust Mean Absolute Deviation |  |
|  |  | Root Mean Squared |  |
|  |  | Skewness |  |
|  |  | Total Energy |  |
|  |  | Uniformity |  |
|  |  | Variance |  |
|  |  |  |  |
| Textural | GLCM | Autocorrelation | 24 |
| features |  | Cluster Prominence |  |
|  |  | Cluster Shade |  |
|  |  | Cluster Tendency |  |
|  |  | Contrast |  |
|  |  | Correlation |  |
| **Classification** | **Method** | **Feature name** | **Count** |
|  |  | Difference Average |  |
|  |  | Difference Entropy |  |
|  |  | Difference Variance |  |
|  |  | Id |  |
|  |  | Idm |  |
|  |  | Idmn |  |
|  |  | Idn |  |
|  |  | Imc1 |  |
|  |  | Imc2 |  |
|  |  | Inverse Variance |  |
|  |  | Joint Average |  |
|  |  | Joint Energy |  |
|  |  | Joint Entropy |  |
|  |  | MCC |  |
|  |  | Maximum Probability |  |
|  |  | Sum Average |  |
|  |  | Sum Entropy |  |
|  |  | Sum Squares |  |
|  |  |  |  |
|  | GLDM | Dependence Entropy | 14 |
|  |  | Dependence Non-Uniformity |  |
|  |  | Dependence Non-Uniformity Normalized |  |
|  |  | Dependence Variance |  |
|  |  | Gray Level Non-Uniformity |  |
|  |  | Gray Level Variance |  |
|  |  | High Gray Level Emphasis |  |
|  |  | Large Dependence Emphasis |  |
|  |  | Large Dependence High Gray Level Emphasis |  |
|  |  | Large Dependence Low Gray Level Emphasis |  |
|  |  | Low Gray Level Emphasis |  |
|  |  | Small Dependence Emphasis |  |
|  |  | Small Dependence High Gray Level Emphasis |  |
|  |  | Small Dependence Low Gray Level Emphasis |  |
|  |  |  |  |
|  | GLRLM | Gray Level Non-Uniformity | 16 |
|  |  | Gray Level Non-Uniformity Normalized |  |
|  |  | Gray Level Variance |  |
|  |  | High Gray Level Run Emphasis |  |
|  |  | Long Run Emphasis |  |
|  |  | Long Run High Gray Level Emphasis |  |
| **Classification** | **Method** | **Feature name** | **Count** |
|  |  | Long Run Low Gray Level Emphasis |  |
|  |  | Low Gray Level Run Emphasis |  |
|  |  | Run Entropy |  |
|  |  | Run Length Non-Uniformity |  |
|  |  | Run Length Non-Uniformity Normalized |  |
|  |  | Run Percentage |  |
|  |  | Run Variance |  |
|  |  | Short Run Emphasis |  |
|  |  | Short Run High Gray Level Emphasis |  |
|  |  | Short Run Low Gray Level Emphasis |  |
|  |  |  |  |
|  | GLSZM | Gray Level Non-Uniformity | 16 |
|  |  | Gray Level Non-Uniformity Normalized |  |
|  |  | Gray Level Variance |  |
|  |  | High Gray Level Zone Emphasis |  |
|  |  | Large Area Emphasis |  |
|  |  | Large Area High Gray Level Emphasis |  |
|  |  | Large Area Low Gray Level Emphasis |  |
|  |  | Low Gray Level Zone Emphasis |  |
|  |  | Size Zone Non-Uniformity |  |
|  |  | Size Zone Non-Uniformity Normalized |  |
|  |  | Small Area Emphasis |  |
|  |  | Small Area High Gray Level Emphasis |  |
|  |  | Small Area Low Gray Level Emphasis |  |
|  |  | Zone Entropy |  |
|  |  | Zone Percentage |  |
|  |  | Zone Variance |  |
|  |  |  |  |
| Wavelet  features | Wavelet decomposition and filtering of first-order and textural features | Eight wavelet decomposition levels: HHH, HHL, HLH, HLL, LHH, LHL, LLH and LLL.  Five filters: exponential, gradient, logarithm, square and square root. | 1144 |
|  |  |  |  |
| Total |  |  | 1245 |

*GLCM* gray level co-occurrence matrix, *MCC* maximal correlation coefficient, *GLDM* gray level dependence matrix, *GLRLM* gray level run-length matrix, *GLSZM* gray level size-zone matrix, *HHH* high-high-high, *HHL* high-high-low, *HLH* high-low-high, *HLL* high-low-low, *LHH* low-high-high, *LHL* low-high-low, *LLH* low-low-high, *LLL* low-low-low

**Supplemental Table 2a** Comparison of liver SUVs on pre-treatment PET/CT between training and validation cohorts

| **Parameter** | **Training cohort**  **(*n* = 100)** | **Validation cohort**  **(*n* = 52)** | ***P* *** |
| --- | --- | --- | --- |
| Liver SUVmean | 1.79 ± 0.30 | 1.82 ± 0.28 | 0.551 |
| Liver SUVmax | 2.51 ± 0.41 | 2.56 ± 0.38 | 0.466 |

*SUV* standardized uptake value, *SUVmean* mean standardized uptake value, *SUVmax* maximum standardized uptake value

* *P* value was calculated by independent *t*-test.

**Supplemental Table 2b** Comparison of liver SUVs between pre-treatment and end-of-treatment PET/CT (*n* = 96)

| **Parameter** | **Pre-treatment**  **PET/CT** | **End-of-treatment**  **PET/CT ^#^** | ***P* *** |
| --- | --- | --- | --- |
| Liver SUVmean | 1.81 ± 0.31 | 1.83 ± 0.32 | 0.661 |
| Liver SUVmax | 2.58 ± 0.39 | 2.61 ± 0.42 | 0.609 |

*SUV* standardized uptake value, *SUVmean* mean standardized uptake value, *SUVmax* maximum standardized uptake value

**^#^** End-of-treatment PET/CT was performed at least 4 weeks after completion of chemotherapy.

* *P* value was calculated by independent *t*-test.

**Supplemental Table 3** Grouping of patients according to the location of tumor bulk

| **Tumor location** | **Training cohort**  **(*n* = 100)** | **Validation cohort**  **(*n* = 52)** | ***P* *** |
| --- | --- | --- | --- |
| Waldeyer’s ring | 10 | 5 | 0.168 |
| Lymph nodes | 50 | 26 |  |
| Spleen | 3 | 7 |  |
| Liver | 1 | 0 |  |
| Bone | 14 | 5 |  |
| Stomach | 14 | 3 |  |
| Intestine | 2 | 3 |  |
| Other extranodal regions | 6 | 3 |  |

* *P* value was calculated by Fisher’s exact test.

**Supplemental Table 4** Numbers of features after repeatability measurement

| **VOI** | **Conventional**  **features** | **Shape**  **features** | **First-order**  **features** | **GLCM** | **GLDM** | **GLRLM** | **GLSZM** | **Wavelet features** | **Total** |
| --- | --- | --- | --- | --- | --- | --- | --- | --- | --- |
| MBV | 3 | 13 | 15 | 21 | 12 | 15 | 16 | 1044 | 1139 |
| TMTV | 3 | 13 | 14 | 17 | 12 | 14 | 14 | 945 | 1032 |

*VOI* volume of interest, *GLCM* gray level co-occurrence matrix, *GLDM* gray level dependence matrix, *GLRLM* gray level run-length matrix, *GLSZM* gray level size-zone matrix, *MBV* metabolic bulk volume, *TMTV* total metabolic tumor volume

**Supplemental Table 5** Results of radiomic feature selection

| **RS** | **Feature name** | **LASSO coefficient** |
| --- | --- | --- |
| **MBV-RS_PFS_** | wavelet.HLL_GLCM_MCC | -0.268 |
|  | gradient_GLRLM_Gray Level Non-Uniformity | 0.143 |
|  | wavelet.LHL_GLSZM_Gray Level Non-Uniformity | 0.106 |
|  | logarithm_GLSZM_Small Area Low Gray Level Emphasis | -0.092 |
| **MBV-RS_OS_** | wavelet.HHH_GLSZM _Gray Level Non-Uniformity | 0.436 |
|  | wavelet.HHH_GLDM_Large Dependence High Gray Level Emphasis | 0.208 |
|  | logarithm_GLSZM_Small Area Low Gray Level Emphasis | -0.058 |
|  | exponential_firstorder_Skewness | -0.041 |
|  | logarithm_GLSZM_Zone Entropy | 0.013 |
| **TMTV-RS_PFS_** | exponential_GLSZM_Gray Level Non-Uniformity | 0.577 |
|  | gradient_GLRLM_Gray Level Non-Uniformity | 0.063 |
|  | wavelet.HHH_GLSZM_Large Area High Gray Level Emphasis | 0.011 |
| **TMTV-RS_OS_** | gradient_GLSZM_Gray Level Non-Uniformity | 0.598 |
|  | wavelet.HHH_GLSZM_Size Zone Non-Uniformity | 0.102 |
|  | wavelet.HHH_GLSZM_Gray Level Non-Uniformity | 0.025 |

*RS* adiomic signature, *LASSO* least absolute shrinkage and selection operator, *MBV* metabolic bulk volume, *PFS* progression-free survival, *HLL* high-low-low, *GLCM* gray level co-occurrence matrix, *MCC* maximal correlation coefficient, *GLRLM* gray level run-length matrix, *LHL* low-high-low, *GLSZM* gray level size-zone matrix, *OS* overall survival, *HHH* high-high-high, *GLDM* gray level dependence matrix, *TMTV* total metabolic tumor volume

**Supplemental Table 6** Univariate cox analyses for PFS and OS

| **Variables** | **PFS** | | |  | **OS** | | |
| --- | --- | --- | --- | --- | --- | --- | --- |
|  | HR | 95% CI | *P* |  | HR | 95% CI | *P* |
| Gender (male/female) | 1.764 | 0.858-3.627 | 0.123 |  | 2.005 | 0.808-4.971 | 0.133 |
| β2-MG (≤ 3/> 3 mg/L) | 2.027 | 1.018-4.036 | 0.045 |  | 1.956 | 0.911-4.197 | 0.085 |
| B symptoms (no/yes) | 2.794 | 1.404-5.560 | 0.003 |  | 2.145 | 0.995-4.625 | 0.052 |
| IPI score (≤ 2/> 2) | 5.788 | 2.508-13.36 | < 0.001 |  | 8.415 | 2.906-24.37 | < 0.001 |
| Cell of origin (GCB/non-GCB) | 1.245 | 0.561-2.760 | 0.590 |  | 1.098 | 0.464-2.598 | 0.831 |
| MBV-RS_PFS_ (≤ 0.01/> 0.01) | 5.975 | 2.461-14.51 | < 0.001 |  | NA | NA | NA |
| MBV-RS_OS_ (≤ -0.14/> -0.14) | NA | NA | NA |  | 9.982 | 3.432-29.03 | < 0.001 |
| TMTV-RS_PFS_ (≤ -0.21/> -0.21) | 7.515 | 3.095-18.24 | < 0.001 |  | NA | NA | NA |
| TMTV-RS_OS_ (≤ -0.28/> -0.28) | NA | NA | NA |  | 6.535 | 2.472-17.27 | < 0.001 |

*PFS* progression-free survival, *OS* overall survival, *HR* hazard ratio, *CI* confidence interval, *β2-MG* β2-microglobulin, *IPI* International Prognostic Index, *GCB* germinal center B-cell like, *MBV* metabolic bulk volume, *RS* radiomic signature, *NA* not applicable, *TMTV* total metabolic tumor volume

**Supplemental Table 7** Multivariate cox analyses for PFS and OS

| **Variables** | **PFS** | | |  | **OS** | | |
| --- | --- | --- | --- | --- | --- | --- | --- |
|  | HR | 95% CI | *P* |  | HR | 95% CI | *P* |
| **With MBV-RS_PFS_** |  |  |  |  |  |  |  |
| MBV-RS_PFS_ (≤ 0.01/> 0.01) | 4.479 | 1.821-11.02 | 0.001 |  | NA | NA | NA |
| IPI score (≤ 2/> 2) | 4.486 | 1.812-11.11 | 0.001 |  | NA | NA | NA |
| β2-MG (≤ 3/> 3 mg/L) | 0.766 | 0.357-1.647 | 0.495 |  | NA | NA | NA |
| B symptoms (no/yes) | 1.631 | 0.784-3.394 | 0.191 |  | NA | NA | NA |
| **With MBV-RS_OS_** |  |  |  |  |  |  |  |
| MBV-RS_OS_ (≤ -0.14/> -0.14) | NA | NA | NA |  | 7.844 | 2.678-22.97 | < 0.001 |
| IPI score (≤ 2/> 2) | NA | NA | NA |  | 6.417 | 2.203-18.69 | < 0.001 |
| **With TMTV-RS_PFS_** |  |  |  |  |  |  |  |
| TMTV-RS_PFS_ (≤ -0.21/> -0.21) | 4.455 | 1.691-11.74 | 0.003 |  | NA | NA | NA |
| IPI score (≤ 2/> 2) | 3.280 | 1.290-8.340 | 0.013 |  | NA | NA | NA |
| β2-MG (≤ 3/> 3 mg/L) | 0.794 | 0.365-1.726 | 0.560 |  | NA | NA | NA |
| B symptoms (no/yes) | 1.322 | 0.618-2.829 | 0.471 |  | NA | NA | NA |
| **With TMTV-RS_OS_** |  |  |  |  |  |  |  |
| TMTV-RS_OS_ (≤ -0.28/> -0.28) | NA | NA | NA |  | 3.528 | 1.265-9.838 | 0.016 |
| IPI score (≤ 2/> 2) | NA | NA | NA |  | 5.098 | 1.660-15.66 | 0.004 |

*PFS* progression-free survival, *OS* overall survival, *HR* hazard ratio, *CI* confidence interval, *MBV* metabolic bulk volume, *RS* radiomic signature, *NA* not applicable, *IPI* International Prognostic Index, *β2-MG* β2-microglobulin, *TMTV* total metabolic tumor volume
